# Inorganic Materials by Atomic Layer Deposition for Perovskite Solar Cells

**DOI:** 10.3390/nano11010088

**Published:** 2021-01-03

**Authors:** Helen Hejin Park

**Affiliations:** Advanced Materials Division, Korea Research Institute of Chemical Technology (KRICT), Daejeon 34114, Korea; hhpark@krict.re.kr

**Keywords:** metal halide perovskite, solar cell materials, atomic layer deposition, photovoltaics

## Abstract

Organic–inorganic hybrid perovskite solar cells (PSCs) have received much attention with their rapid progress during the past decade, coming close to the point of commercialization. Various approaches in the process of PSC development have been explored with the motivation to enhance the solar cell power conversion efficiency—while maintaining good device stability from light, temperature, and moisture—and simultaneously optimizing for scalability. Atomic layer deposition (ALD) is a powerful tool in depositing pinhole-free conformal thin-films with excellent reproducibility and accurate and simple control of thickness and material properties over a large area at low temperatures, making it a highly desirable tool to fabricate components of highly efficient, stable, and scalable PSCs. This review article summarizes ALD’s recent contributions to PSC development through charge transport layers, passivation layers, and buffer and recombination layers for tandem applications and encapsulation techniques. The future research directions of ALD in PSC progress and the remaining challenges will also be discussed.

## 1. Introduction

As solar energy is the most abundant energy resource available to humankind, various photovoltaic (PV) technologies have been investigated by researchers over the past years with the common motivation to efficiently and cost-effectively convert solar energy into electricity. Organic–inorganic hybrid perovskite solar cells (PSCs) make up an exciting field of PV technology research due to its speedy progress during the past decade arriving close to the point of commercialization. Although PSCs have shown promising results in terms of theirs rapid progress in power conversion efficiency, already at 25.5% for unit cells [1], they are different from other PV technologies as they display other challenges especially towards long-term stability of the working devices from both external and internal factors. Device deterioration from internal factors include ion migration from the perovskite layer and from dopants of hole transport layers diffusing out into the perovskite, whereas external factors include deterioration from exposure to light, elevated temperatures, and air (moisture and oxygen) [2,3,4,5,6,7,8,9,10]. In order for the actual commercialization of PSCs to occur, optimization of the following aspects will be required: high efficiency, long-term stability, possible large-area coating techniques, consideration of semitransparent and tandem applications, and nontoxicity and low-cost of the materials and fabrication processes.

Atomic layer deposition (ALD) is a powerful tool for growing reproducible conformal pinhole-free high-quality thin-films of inorganic materials. ALD has the advantage of precise fine control of the film thickness and materials properties, such as stoichiometry, morphology, and doping [11,12,13,14,15]. While chemical vapor deposition (CVD) and physical vapor deposition (PVD), including thermal evaporation, e-beam evaporation, molecular beam epitaxy, pulsed laser deposition, and sputtering, have been popular deposition methods in industry, ALD has gained much attention with ultra-thin or pinhole-free conformal coating becoming more important. Film formation by PVD methods are usually by direct transport of the source to the substrate through the gas phase, while film formation by CVD involves chemical reaction between mixed gas sources on a substrate. In comparison to PVD and CVD, ALD is a low-vacuum and low-temperature deposition technique, which allows excellent conformal and uniform coating of 3D structures and precise control of film thickness due to its self-limiting chemisorption of precursors during the ALD cycle. However, such high precision leads to high usage of the ALD precursors and co-reactants, which lead to waste of materials, as approximately 60% of the precursor pulse is wasted in the ALD growth process. Another disadvantage is the cost-effectiveness of ALD in commercial use due to its low deposition rates. Such challenges have been partially overcome by spatial-ALD [16,17].

Due to its possibilities for deposition over large areas at low temperatures, ALD has already shown to be useful in various other applications, such as microelectronics, batteries, and other leading PV technologies, including crystalline silicon and copper indium gallium diselenide (CIGS) thin-film solar cells [18,19,20,21,22]. Thus, ALD is a highly attractive tool to fabricate components of layers in PSCs, and its contribution to the advancement of PSC should be fully utilized and explored. This review article will summarize and highlight cases where ALD has been applied to the development of PSCs and provide insight into the current challenges and future of ALD in the development of PSCs.

**Figure 1:**
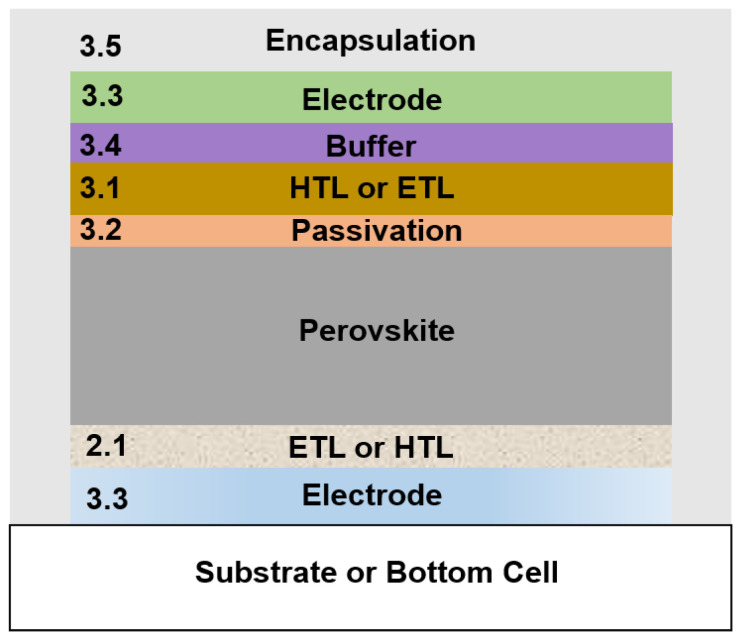
Schematic graphic for the overall concept in the application of atomic layer deposition (ALD) in perovskite solar cells (PSCs). The corresponding section numbers in this review are denoted for the incorporation of ALD for each layer.

As the perovskite and organic transport layers are particularly susceptible to elevated temperatures and exposure to certain ALD precursors under low vacuum, this review will be divided into parts focusing on the developments below and above the perovskite layer. As summarized in the schematic graphic in Figure 1, ALD can be applied in PSCs for the electrodes, charge transport layers (CTLs) below and above the perovskite, passivation layer, buffer layer, and encapsulation. The corresponding section numbers in this review for the application of ALD for each layer are shown in the schematic. First, in Section 2, we investigate the cases where ALD processes were adopted for the deposition of layers under the perovskite layer as electron transport layers (ETLs, Section 2.1.1) and hole transport layers (HTLs, Section 2.1.2).

In Section 3, we explore the developments made for ALD utilized for layers above the perovskite absorber as CTLs (Section 3.1), passivation or barrier layers (Section 3.2), recombination layers or bottom electrodes (Section 3.3), buffer layers in semitransparent and tandem applications (Section 3.4), and encapsulation (Section 3.5). The efforts of ALD in halide perovskite light emitting diodes will also be discussed (Section 4). Variations of ALD techniques, such as pulsed-chemical vapor deposition (pulsed-CVD) and spatial ALD (s-ALD), will be further discussed in Section 5, and conclusions and future outlooks will be discussed in Section 6.

## 2. ALD below the Perovskite Layer

### 2.1. Charge Transport Layers below the Perovskite Layer

Despite the many advantages of ALD, there are certain issues that get introduced when dealing with PSCs as organic charge transport layers and the perovskite absorber are susceptible to damage from exposure to elevated temperatures and certain ALD precursors in the presence of low vacuum levels of approximately hundreds of millitorrs. However, this is not as much of a problem when applying ALD to grow films below the perovskite layer. Various studies in the literature of ALD films incorporated into PSCs for layers below the perovskite absorber are summarized in Table 1. We will first begin with discussing charge transport layers (including ETLs and HTLs) below the perovskite layer and then move onto layers above the perovskite absorber in Section 3.

#### 2.1.1. Electron Transport Layers

**Figure 2:**
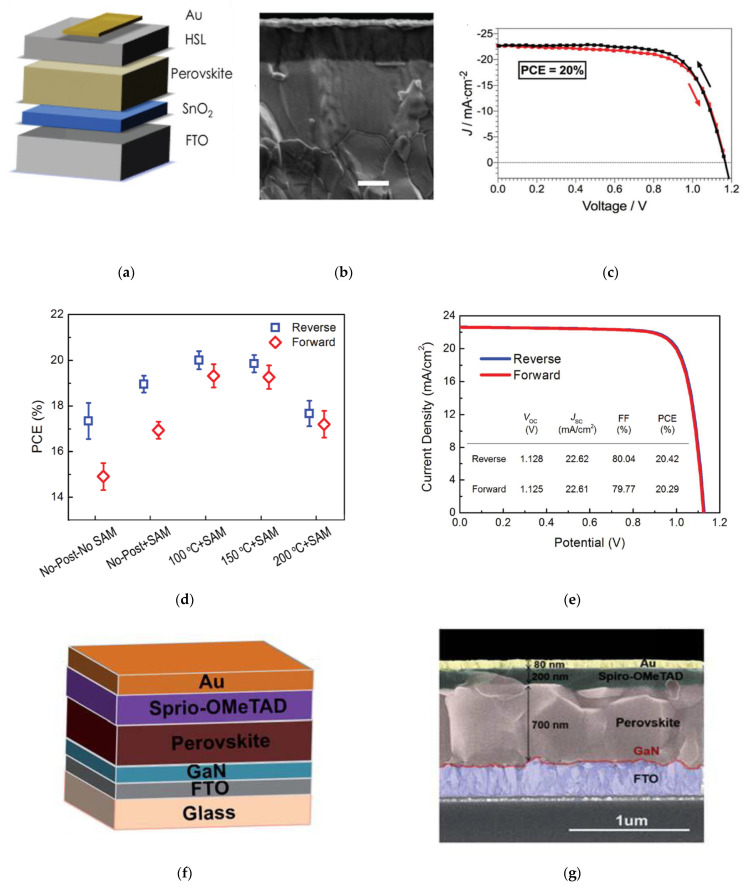
Incorporation of atomic layer deposition (ALD) processes for SnO_2_ as electron transport layer (ETL): (**a**) Schematic of device configuration; (**b**) Cross-sectional scanning electron microscopy (SEM) image of the planar SnO_2_-based PSC (scale bar is 200 nm); (**c**) Illuminated current density vs. voltage scans for PSC with 15 nm of ALD SnO_2_. Reproduced from [24], with permission from the Royal Society of Chemistry, 2017. Incorporation of ALD processes for SnO_2_ as ETL in PSC with self-assembly monolayer (SAM) treatments: (**d**) Comparison of power conversion efficiency (PCE) between reverse and forward voltage scan for varying post-annealing and SAM treatments; (**e**) Illuminated current density vs. voltage (J–V) of optimal device. Reproducedfrom [25], with permission from Wiley, 2017. Incorporation of ALD processes for GaN as ETL in PSC: (**f**) Solar cell device configuration; (**g**) Cross-sectional SEM image of device. Reproduced from [30], with permission from the Royal Society of Chemistry, 2019.

The most commonly used materials for ETLs in *n*-*i*-*p* structured PSCs, include mesoporous titanium dioxide (TiO_2_) for mesoscopic structures, and tin dioxide (SnO_2_) by chemical bath deposition (CBD) or spin-coating nanoparticles for planar structures [23]. Various laboratories have explored alternatively growing ETLs by ALD using materials such as TiO_2_, SnO_2_, zinc oxide (ZnO), and niobium oxide (Nb_2_O_5_). Perovskite solar cells with ALD SnO_2_ using deposition temperatures in the range of 100–120 °C have been demonstrated by tetrakis-dimethyl-amine tin (TDMASn) and ozone (O_3_) or oxygen (O_2_) plasma, as the tin and oxygen precursors, respectively, reaching power conversion efficiencies (PCE, *η*) over 20% [24]. ALD SnO_2_ without any post-treatments or additional electron transporting materials result in a PCE of 20% when using a triple cation perovskite followed by 2,2′,7,7′-tetrakis-(*N*,*N*-di-4-methoxyphenylamino)-9,9′-spirobifluorene (spiro-OMeTAD) and gold (Au), as shown in Figure 2a–c. ALD SnO_2_ followed by fullerene-self-assembly monolayer (C_60_-SAM) is reported to effectively improve the charge collection at the ETL/perovskite interface, the current density vs. voltage (*J*–*V*) scan hysteresis is still an issue [25]. Post-annealing ALD SnO_2_ at 100 °C in air resulted in improved electron mobility of SnO_2_, and consequently enhanced the PCE and reduced the *J–V* hysteresis (Figure 2d), resulting in PCE over 20% (Figure 2e).

Some reports have also demonstrated ALD *n*-type materials to be effective in surface treatment of the transporting conducting oxide (TCO)/ETL or ETL/perovskite interfaces, instead of completely replacing the conventional nanoparticle SnO_2_ (*np*-SnO_2_) or bilayer TiO_2_ structures, which consists of a compact TiO_2_ layer (*c*-TiO_2_) followed by a mesoporous TiO_2_ (*mp*-TiO_2_) layer. Previous studies investigated ALD TiO_2_ mostly in between the TCO, usually indium tin oxide (ITO) or fluorine-doped tin oxide (FTO), and *mp*-TiO_2_ or *np*-SnO_2_ or in between the ETL and perovskite layers [26].

For ALD TiO_2_ incorporation into PSCs, titanium precursors, such as titanium tetrachloride (TiCl_4_), tetrakis-dimethyl-amino titanium (TDMAT), titanium isopropoxide (TTIP), cyclopentadienyl alkylamido (Ti(CpMe)(NMe_2_)_3_), have been investigated with H_2_O as the oxygen precursor [27]. In the case of mesoporous antimony-doped tin oxide nanorod arrays as the ETL in PSCs, a dense 10-nm TiO_2_ layer by ALD resolved the issue of uneven growth of the perovskite absorber layer on a rough surface, resulting in highly smooth, dense, and crystallized perovskite films in solar cell devices with PCEs above 20% [28]. Similarly, a thin film (<2 nm) of zinc sulfide (ZnS) by ALD has been incorporated in between the *mp*-TiO_2_/perovskite interface in PSCs to improving charge extraction properties and reducing interface recombination, resulting in efficiencies over 19% with negligible hysteresis [29].

Without the assistance of any additional solution-processed ETLs, gallium nitride (GaN) by ALD was incorporated as the ETL in *n*-*i*-*p* PSCs, using triethylgallium (TEG) as the gallium precursor, and a high-purity Ar/N_2_/H_2_ (1:3:6, 99.999%) plasma as the nitrogen source [30] (Figure 2f,g). Despite the conduction-band-minimum mismatch of 0.59 eV, 5 nm of ALD GaN between the FTO/perovskite interface improved the PCE from 10.4% to 15.2%.

#### 2.1.2. Hole Transport Layers

While solution-processable poly(triarylamine) (PTAA), poly(3,4-ethylenedioxythiophene) polystyrenesulfonate (PEDOT:PSS), nickel oxide (NiO) nanoparticles, and copper thiocyanate (CuSCN) are the common HTL materials by solution processing [50,51,52], several groups have investigated replacing these HTL materials with ALD-grown inorganic materials, including NiO*_x_*, vanadium oxide (VO*_x_*), and iridium-doped titanium dioxide (TiO_2_-IrO*_x_*).

**Figure 3:**
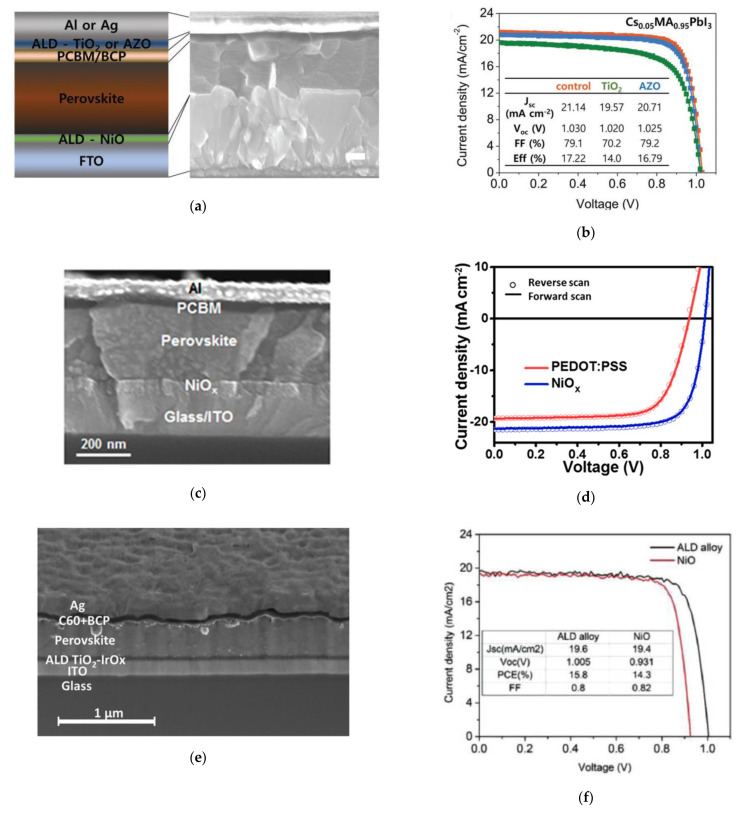
Incorporation of ALD processes for NiO, TiO_2_, and aluminum-doped zinc oxide (AZO) in PSC: (**a**) Schematic of solar cell device stack and cross-sectional scanning electron microscopy image of the PSC device; (**b**) Illuminated *J–V* scans comparing PSCs with ALD TiO_2_ and ALD AZO. Reproduced from [47], with permission from Wiley, 2018. Incorporation of atmospheric pressure spatial ALD (s-ALD) processes for NiO*_x_* as hole transport layer (HTL) in PSC: (**c**) Cross-sectional SEM image of the PSC device; (**d**) Illuminated *J–V* scans comparing PSCs with the conventional HTL, poly(3,4-ethylenedioxythiophene) polystyrenesulfonate (PEDOT:PSS), and s-ALD NiO*_x_* (right). Reproduced from [45], with permission from the American Chemical Society, 2018. Incorporation of ALD processes for TiO_2_-IrO*_x_* as HTL in PSC: (**e**) Cross-sectional scanning electron microscopy image of PSC device; (**f**) Illuminated current density vs. voltage comparing PSCs with ALD TiO_2_-IrO*_x_* and nanoparticle NiO. Reproduced from [43], with permission from Wiley, 2018.

The solution-processed nickel oxide HTL was successfully replaced with ALD NiO using bis(1-dimethylamino-2-methyl-2-butanolate)nickel (Ni(dmamb)_2_) and O_3_ as the nickel and oxygen precursors, respectively, resulting in efficiencies over 17% for *p*-*i*-*n* structured PSCs (Figure 3a,b) [47].

Researchers have also successfully demonstrated *p*-*i*-*n* structured PSCs with NiO*_x_* by an atmospheric pressure spatial ALD (s-ALD) system using bis(methylcyclopentadienyl)nickel(II) (Ni(MeCp)_2_) and oxygen (O_2_) gas as the nickel and oxygen precursors, respectively (Figure 3c). Such rapid production of high-quality NiO*_x_* HTLs resulted in PCEs over 17% and fill factors over 80% with negligible hysteresis [45] (Figure 3d). The high-uniformity of s-ALD NiO*_x_* films enabled perovskite films with improved intrinsic electronic quality and efficient collection of charge carriers, resulting in PSC devices with improved open-circuit voltage (*V_OC_*) and reduced interfacial trapping.

While TiO_2_ is a well-known ETL in photovoltaic devices, alloying TiO_2_ with iridium oxide (IrO*_x_*) by ALD was demonstrated to exhibit a high work function appropriate for hole extraction [43], as shown in Figure 3e. Such an ALD alloy as an HTL in PSCs was deposited with TDMAT, 1-ethylcyclopentadienyl-1,3-cyclohexadiene iridium(I) ((EtCp)Ir(CHD)), and O_3_ as the titanium, iridium, and oxygen precursors, respectively. By adjusting the IrO*_x_* contents, which is easily done by modifying the ALD sequence, TiO_2_-IrO*_x_* alloys as the HTL in *p*-*i*-*n* structured PSCs resulted in efficiencies over 15%, fill factors of 80%, and *V_OC_*’s over 1 V (Figure 3f).

## 3. ALD above the Perovskite Layer

### 3.1. Charge Transport Layers above the Perovskite Layer

Deposition condition restrictions arise in the case of utilizing ALD to deposit layers on top of the perovskite layer due to the perovskite and/or organic HTL’s susceptibility to thermal energy, moisture, and exposure to certain ALD precursors (including H_2_O) and low vacuum levels for extended periods of time. Various studies in the literature of ALD films incorporated into PSCs for layers above the perovskite absorber are summarized in Table 2, which include CTLs above the perovskite absorber, passivation (or barrier) layers directly on top of the perovskite, recombination layers in all-perovskite tandem applications, and buffer layers in semitransparent and tandem applications.

The steps involved in a single ALD cycle process involves a metal precursor pulse exposed to the substrate, followed by a purging step with a carrier gas, followed by the co-reactant pulse exposed to the substrate, followed by another purging step with a carrier gas. The ALD cycle is repeated until the desired thickness is achieved. Deionized water has shown to be the least damaging to the perovskite active layer, among the various co-reactants for the oxygen precursor (H_2_O, ozone, and O_2_ plasma). A study showed that ALD Al_2_O_3_ by trimethylaluminum (TMA) and ozone resulted in a complete loss of the MAPbI_3-*x*_Cl*_x_* phase, and bleached the perovskite layer [53], while another study showed that O_2_ plasma processes resulted in partial degradation of MAPbI_3_ to PbI_2_ even at a very low deposition temperature of 30 °C [54]. Based on previous reports, each pulse step (the metal precursor and the co-reactant) influences the perovskite active layer in somewhat contradicting ways. While a study reported to not observe any degradation of the perovskite after exposure of repeated pulses of TMA and H_2_O at 80 °C, several studies reported the loss of nitrogen, implying etching of MA^+^ from the perovskite active layer [55]. A study found that TMA partial pressures of 0.1 Torr can etch MAPbI_3_ at 75 °C, and observed continual mass loss of perovskite at high TMA exposures of 3 Torr at 25 °C [56], suggesting that variations in ALD process parameters result in very different perovskite surfaces, which may explain the discrepancies in literature. For ALD SnO_2_ growth by TDMASn and H_2_O, a study reported unaltered perovskite surface composition and bulk crystallinity after a >11 Torr· exposure of TDMASn at 120 °C [57]. However, another study observed removal of FA^+^ from the perovskite surface and formation of PbI_2_ after exposure to 60 cycles of TDMASn and H_2_O [58]. In addition, these results indicated that the TDMASn has a stronger effect on the perovskite degradation compared to H_2_O. Based on these previous results, the general consensus appears to be that deposition temperatures below 100 °C and H_2_O co-reactants are preferred in avoiding etching of the perovskite surface and bulk degradation.

Although directly on top of the perovskite absorber layer, passivation layers only require 1 nm or less, so the duration of the absorber material to be exposed to elevated temperatures, ALD precursors, and low vacuum level is rather short (within 10 min). However, as charge transport layers normally require larger thicknesses around 40 nm, this especially becomes an issue if the thick CTL is directly on top of the perovskite absorber. Thus, there are approaches where the perovskite absorber is protected with various other layers to avoid direct exposure of the perovskite surface to the ALD processing conditions. To avoid direct exposure of ALD precursors to the perovskite active layer, an organic ETL, such as C_60_ or [6,6]-phenyl C_61_ butyric acid methyl ester (PCBM), or organic HTL, such as PTAA, are typically used as an interfacial layer to protect the perovskite from surface etching and/or bulk degradation [59,60]. Based on XRD analysis, approximately 50 nm of PTAA was sufficient enough to protect the underlying perovskite active layer from ALD processing damage, whereas direct CuO*_x_* deposition on top of the bare perovskite surface resulted in bulk degradation.

With ALD aluminum-doped zinc oxide (AZO) in between PCBM/BCP and the top electrode, the degradation of the perovskite absorber from external water and evaporation of methylammonium (MA) was retarded at elevated temperatures of 85 °C. The high conductivity of AZO also enabled efficient charge extraction from [6,6]-phenyl C_61_ butyric acid methyl ester (PCBM) transferred to the top silver (Ag) electrode. ALD AZO was deposited from trimethylaluminum (TMA), diethylzinc (DEZ), and H_2_O as the aluminum, zinc, and oxygen precursors, respectively.

### 3.2. Passivation or Barrier Layers

A very thin layer (< 1 nm) directly on top of the perovskite absorber, known as the passivation or barrier layer, has been shown to be very effective in not only improving the solar cell device performance, through improvement in open-circuit voltage and fill factor, but also in improving the device stability [61]. Such improvement in operational stability can be explained via providing a barrier between the perovskite and charge transport layer, or surface passivation of the perovskite layer. While similar surface passivation concepts have been demonstrated by forming a two-dimensional perovskite on top of the three-dimensional perovskite layer by solution processing, some common barrier layers have also been investigated by ALD resulting in improved device stability to moisture and light. Several groups have demonstrated barrier layers by ALD using aluminum oxide (Al_2_O_3_) and zirconium oxide (ZrO_2_).

**Figure 4:**
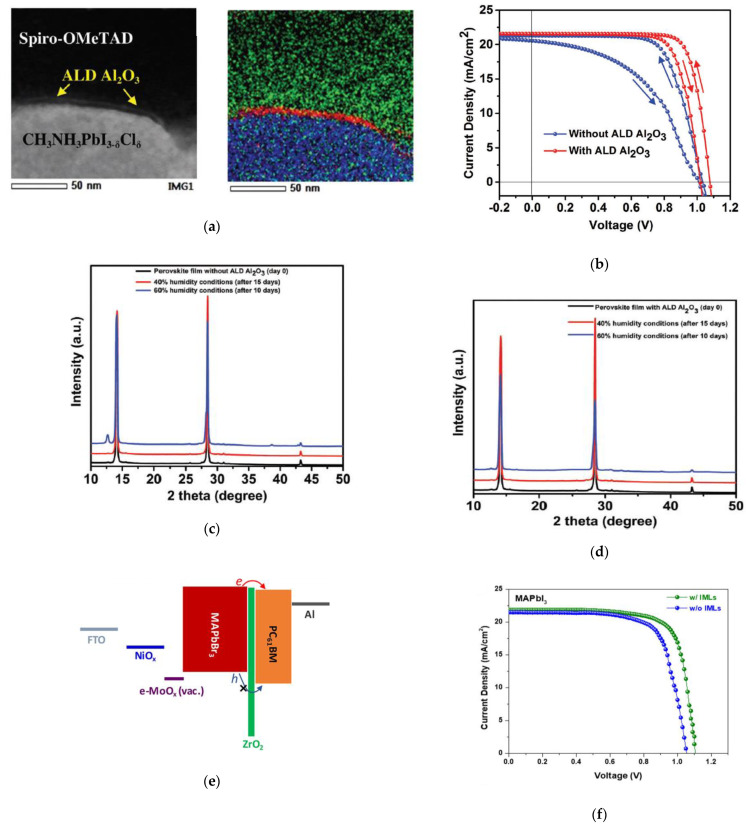
Incorporation of ALD Al_2_O_3_ as passivation layer in PSC: (**a**) high angle annular dark field (HAADF) scanning transmission electron microscopy (TEM) image of the perovskite/Al_2_O_3_/spiro-OMeTAD interface, and the corresponding overlapping elemental mapping image; (**b**) Illuminated *J*-*V* scans comparing PSCs without and with Al_2_O_3_; (**c**) X-ray diffraction (XRD) scans of perovskite films without Al_2_O_3_; (**d**) X-ray diffraction (XRD) scans of perovskite films with Al_2_O_3_. Reproduced from [62], with permission from the Royal Society of Chemistry, 2017. Incorporation of ALD ZrO_2_ as barrier layer in PSC: (**e**) Illuminated current density vs. voltage comparing PSCs without and with the ALD Al_2_O_3_ barrier layer; (**f**) Illuminated current density vs. voltage comparing PSCs with ALD TiO_2_-IrO*_x_* and nanoparticle NiO. Reproduced from [74], with permission from Wiley, 2018.

Inserting a ultra-thin (<1 nm) Al_2_O_3_ passivation layer in between the perovskite absorber and spiro-OMeTAD in the conventional *n*-*i*-*p* structured PSC (Figure 4a), resulted in improved device performance through enhanced *V_OC_* and fill factor [62] (Figure 4b). The Al_2_O_3_ passivation layer not only improved the PCE of the PSC, but also resulted in reduced hysteresis and stabilized the device against high humidity. Based on X-ray diffraction (XRD) scans of perovskite films after exposure to humidity, samples without Al_2_O_3_ resulted in the appearance of a PbI_2_ (001) diffraction peak as a result of decomposition of MAPbI_3_ (Figure 4c), whereas samples with Al_2_O_3_ did not show the appearance of this peak (Figure 4d). Photovoltaic performance monitoring after exposure to humid conditions also resulted in more stable PSCs for devices with the Al_2_O_3_ passivation layer.

Investigation of ZrO_2_ passivation (Figure 4e) also resulted in enhanced PCEs from improved *V_OC_* values for *p*-*i*-*n* structured PSCs (Figure 4f). MAPbI_3_ based PSCs showed an enhancement in *V_OC_* by 0.1 eV, while MAPbBr_3_ based PSCs showed an enhancement in *V_OC_* by 0.5 V with insertion of the ZrO_2_ passivation layer at the perovskite/ETL interface. Shelf-stability of devices with and without ZrO_2_ also showed substantial improvement in stability [74].

**Figure 5:**
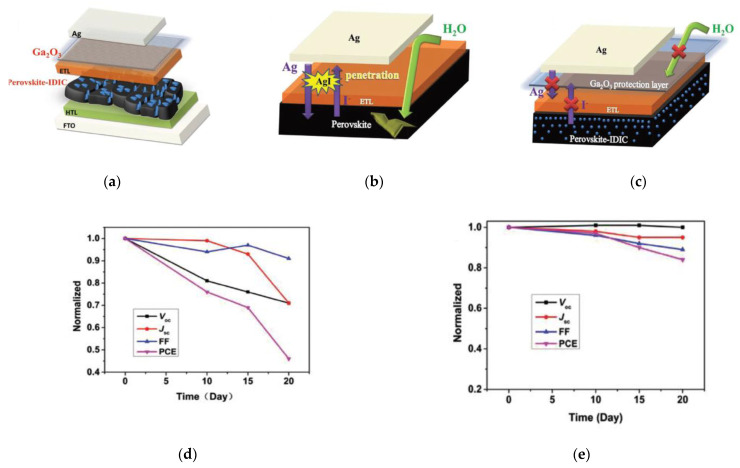
Incorporation of ALD Ga_2_O_3_ as barrier layer between the ETL and top electrode in the PSC: (**a**) Schematic of the device configuration; (**b**) Illustration of degradation mechanism in the case without Ga_2_O_3_; (**c**) Illustration of the protection effect from degradation in the case with Ga_2_O_3_; (**d**) Normalized photovoltaic performance parameters as a function of ambient storage time for PSCs without Ga_2_O_3_; (**e**) Normalized photovoltaic performance parameters as a function of time for PSCs with Ga_2_O_3_. Reproduced from [65], with permission from Wiley, 2018.

Passivation or protection can also be performed at the CTL/top metal contact interface. A thin (<2 nm) inorganic wide bandgap material gallium oxide (Ga_2_O_3_) by ALD was inserted in between the ETL and top metal contact, silver (Ag), as shown in Figure 5a [65]. Due to Ag and iodine ion diffusion, formation of AgI results in degraded PSC device performance, which is a well-known degradation mechanism (Figure 5b). The insertion of Ga_2_O_3_ results in stabilized devices from preventing formation of AgI, as illustrated in Figure 5c. Such a Ga_2_O_3_ protection layer provides a barrier from the penetration of moisture and hinders the corrosion mechanism from the top Ag electrode, as shown in the normalized performance parameters as a function of ambient storage time for PSCs without and with the Ga_2_O_3_ protection layer (Figure 5d,e). Furthermore, insertion of this protection layer promotes suppressed carrier recombination, decreased current leakage, and improved interfacial contact.

### 3.3. Recombination Layers in Tandem Applications

Recombination layers in tandem applications are required to be conductive with high infrared transparency to electrically and optically integrate to top and bottom solar cells. Aluminum-doped ZnO (AZO) has been one of the commonly explored recombination materials by ALD to replace the conventional sputtered indium tin oxide (ITO) recombination material. Incorporation of an ALD AZO recombination layer into all-perovskite monolithic tandems has been previously demonstrated [63,75]. Recombination layers are critical in monolithic two-terminal tandems in electrically and optically integrating the top and bottom subcells.

**Figure 6:**
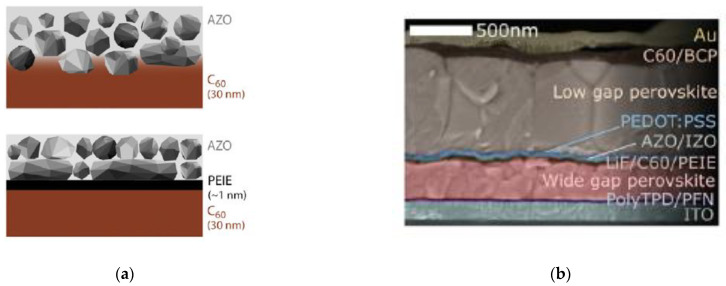
Incorporation of ALD AZO as recombination layer in all-perovskite tandem solar cell: (**a**) Schematic of AZO without and with poly(ethylenimine) ethoxylated (PEIE) nucleation layer; (**b**) Cross-sectional SEM of solar cell stack; Reproduced from [63], with permission from Elsevier, 2019.

It is also critical to develop fabrication processes of the recombination layer that does not damage the bottom subcell, but also make sure the recombination layer is not damaged from the fabrication processes for the top subcell. Previous studies report that a nucleation layer of an ultra-thin polymer, poly(ethylenimine) ethoxylated (PEIE), enables nucleation of a conformal low-conductivity AZO layer by ALD (Figure 6a–b). This method is stated to allow ALD-grown recombination layers which reduce shunting and solvent degradation from solution processing of the layers from the top cell.

### 3.4. Buffer Layers in Semitransparent and Tandem Applications

**Figure 7:**
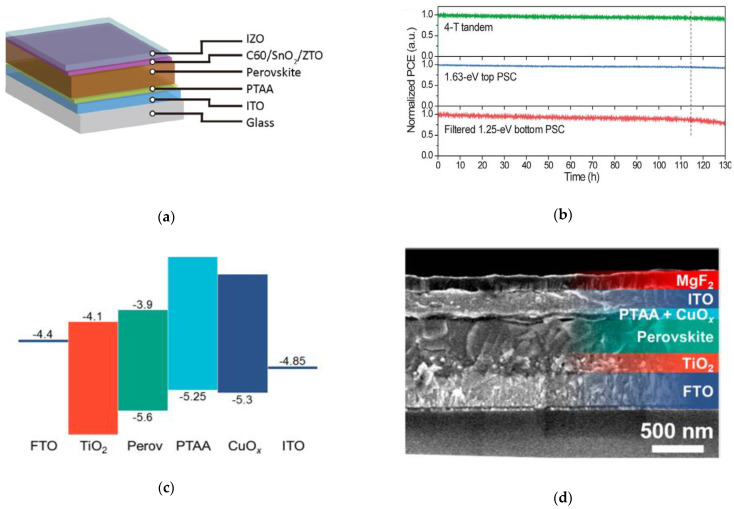
Incorporation of ALD SnO*_x_* and zinc tin oxide (ZTO) in perovskite tandem devices: (**a**) Device stack configuration of the top perovskite cell; (**b**) Normalized PCE as a function of time for the various PSC devices. Reproduced from [59], with permission from the American Association for the Advancement of Science, 2019. Incorporation of atmospheric-pressure chemical vapor deposition (AP-CVD) of CuO*_x_* as a buffer layer in semitransparent PSC: (**c**) Schematic of band alignment of the device; (**d**) Cross-sectional SEM image of the device stack. Reproduced from [64], with permission from the American Chemical Society, 2020.

Compared to opaque devices with a metal top contact, semitransparent and tandem applications require a semitransparent top contact to replace the opaque metal top contact. The most common transparent electrode technique used is a transparent conducting oxide (TCO), such as ITO and indium zinc oxide (IZO), by sputtering. However, this usually requires a buffer layer in below the sputtered TCO, to protect the underlying organic CTL from sputtering damage during the TCO processing. Commonly used sputter buffer layers in *p*-*i*-*n* structured perovskite top cells in tandem applications are SnO_2_ [66] or SnO_2_ followed by zinc tin oxide (ZTO) [59] by ALD to further improve the band alignment at the buffer/TCO interface (Figure 7a), resulting in stable semitransparent PSC under 1-SUN illumination (Figure 7b). Thermally evaporated molybdenum oxide (MoO*_x_*) has been the standard buffer layer in semitransparent *n*-*i*-*p* PSCs, however, it suffers from poor air stability [76]. ALD copper oxide (CuO*_x_*) and vanadium oxide (VO*_x_*) have also been reported as buffer layers in semitransparent PSCs [72,73]. Growth methods by pulsed-chemical vapor deposition (pulsed-CVD) [60] or atmospheric-pressure chemical vapor deposition (AP-CVD) [64] have been reported for CuO*_x_* buffer layers in *n*-*i*-*p* structured semitransparent PSCs. CuO*_x_* films by AP-CVD resulted in high mobilities over 4 cm^2^/V·s, and semitransparent PSCs with these buffer layers resulted in PCEs over 16% (Figure 7c,d) [64].

### 3.5. Encapsulation

Encapsulation is required for most PSCs to protect the layers from external environmental factors, such as oxygen and moisture. Several reports demonstrated successful encapsulation of PSC devices by ALD single materials or nanolaminates of multiple stacks of alternating materials by ALD and/or organic materials. For example, encapsulated semitransparent PSC devices with a bilayer of 50-nm Al_2_O_3_-coated polyethylene terephthalate (PET) resulted in stable devices based on storage in ambient air for over 45 days [55].

## 4. ALD in Perovskite Light Emitting Diode Applications

The usage of ALD in another halide perovskite related field is perovskite-based light emitting diodes (LEDs), in which the deposition control has a great impact on the device performance. A study demonstrated that ZnO can be directly deposited on top of a green-emitting methylammonium lead bromide (MAPbBr_3_) perovskite by spatial-ALD, and by replacing the oxidant H_2_O with oxygen gas. In this study, the LED device had a structure of ITO/PEDOT:PSS/MAPbBr_3_/ZnO/Ca/Ag, and ZnO was deposited in open air onto the perovskite at 60 °C for 3 min, and Mg was incorporated into ZnO to produce Zn_1-*x*_Mg*_x_*O to reduce the electron injection barrier with the perovskite [77]. Another study demonstrated that ZnO can be deposited by ALD in the LED device configuration of ITO/PEDOT:PSS/CsPbBr_3_/ZnO/Ag, by passivating the CsPbBr_3_ with polyethyleneimine ethoxylated (PEIE) dissolved in chlorobenzene to facilitate the growth of ALD ZnO [78]. The hydroxyl groups of PEIE served as surface sites, which reacted with the Zn precursor, DEZ, during the ALD process and allowed ZnO to be deposited on top of the perovskite layer without damage.

## 5. Variations of ALD

While ALD has many advantages, such as accurate control of stoichiometry and thickness with excellent reliability, for certain layers, especially thicker layers (over about 15 nm) above the perovskite absorber, extended duration under exposure to elevated temperatures, certain ALD precursors, and low vacuum, can result in detrimental effects on the perovskite and/or organic CTL [54]. Most ALD processes in PSCs are generally desired to be deposited at low temperatures (<100 °C) if possible to minimize thermally induced stress. In regards to damage due to exposure from ALD precursors, there have been studies showing reduction of stretching and bending modes of N–H with increasing ALD Al_2_O_3_ cycles, based on in situ infrared spectroscopy, which implies loss of nitrogen from etching of the MA^+^ in the perovskite lattice [55]. Thus, variations from the conventional ALD are required to minimize deposition time and exposure to degradation sources.

Some common examples are pulsed-CVD [60], AP-CVD [64], and s-ALD [45,79]. Pulsed-CVD involves reducing the carrier purging step during the ALD sequence and pulsing the ALD precursors simultaneously, instead of separately, to reduce the deposition time [80]. From such variation to the conventional ALD method, pulsed-CVD growth methods can reduce the overall deposition time by over an order of magnitude. In the case of atmospheric-pressure spatial-ALD methods, vapors of precursors are carried through gas lines to the reactor head and flow out of separate channels. Here, metal precursors and co-reactant channels are separated by inert gas channels, to prevent precursors reacting above the substrate, and the heated moving substrate is cycled below the gas head and channels [45]. Some labs reported the use of s-ALD of NiO and SnO_2_ for the HTL and ETL, respectively. A rapid-vapor phase deposition method, or AP-CVD methods have also shown to be successfully incorporated for buffer layers in semitransparent PSC devices.

The advantages and disadvantages of conventional ALD and its variations are summarized in Table 3. While conventional ALD methods have advantages of conformal pinhole-free uniform coating of ultra-thin films, there are disadvantages, such as very slow growth rates and the need for medium vacuum levels. Pulsed-CVD compensates for improving the slow growth rates of the conventional ALD method by cutting down on the purging times. However, conformal coating for complex nanostructures and ultra-thin depositions by pulsed-CVD are not as good as the conventional ALD methods. By moving the substrate between different precursor zones, spatial-ALD does not require any vacuum and has much faster growth rates compared to the conventional ALD method. However, conformal coating by s-ALD is not as good as conventional ALD, and there are limited available precursors since there are issues with sensitivity to ambient oxygen and moisture when processed in air [17].

## 6. Summary and Future Perspectives

In summary, we have reviewed the various selected previous studies on utilizing ALD in perovskite solar cell research. Separating ALD incorporation below and above the perovskite absorber layer in the device configuration, there are still many challenges remaining for especially ALD films above the perovskite absorber layer. ALD layers below the perovskite absorber involve ETL and HTL materials, whereas ALD incorporation of layers above the perovskite absorber involve passivation layers at the perovskite surface, barrier/protection layers at the CTL/top metal contact interface, recombination layers in all-perovskite tandems, buffer layers in semitransparent and tandem applications, and encapsulation layers to improve the device stability from external degradation factors.

ALD is definitely a powerful tool in depositing high-quality dense pinhole-free inorganic materials with excellent reproducibility and easy control of material properties, including stoichiometry, doping, and electrical/optical properties. In order for ALD to be utilized to its full potential in the development of perovskite photovoltaics, there are still several issues to overcome, such as elevated temperatures, damage from ALD precursors, and long deposition times. Semitransparent and tandem solar cells will become a promising entry to the solar PV industry to cost-effectively enhance solar cell efficiencies. Considering such commercialization aspects, ALD and variations of ALD, such as s-ALD, pulsed-CVD, and AP-CVD will contribute to perovskite PV technologies requiring large-area coatings and highly-efficient and stable semitransparent and tandem applications, along with other applications including flexible electronic devices.

## Figures and Tables

**Table 1:** Summary of literature on ALD-grown layers below the absorber in perovskite solar cells.

Material	Precursors	Temp. (°C)	Application/Structure	Device Stack	*J_SC_* (mA/cm^2^)	*V_OC_* (V)	*FF* (%)	*η* (%)	Institute, Year [Ref]
SnO_2_	TDMASn + O_3_	118	ETL/ *n*-*i*-*p*	FTO/SnO_2_ (15 nm)/FA_0.85_MA_0.15_Pb(I_0.85_Br_0.15_)_3_/spiro-OMeTAD/Au	21.3	1.14	74.0	18.4	EPFL, 2015 [31]
SnO_2_	TDMASn + O_3_	100–120	ETL/ *n*-*i*-*p*	FTO/*d*-TiO_2_/SnO_2_/FA_0.85_MA_0.15_Pb(I_0.85_Br_0.15_)_3_/PTAA/Au	22.7	1.13	78.0	20.0	EPFL, 2018 [32]
SnO_2_	TDMASn + O_3_	118	ETL/ *n*-*i*-*p*	FTO/SnO_2_ (15 nm)/Rb1(FA_0.83_MA_0.17_)99Pb(I_0.83_Br_0.17_)_3_/spiro-OMeTAD/Au	23.0	1.17	71.0	20.0	EPFL, 2017 [24]
SnO_2_	TDMASn + O_2_ Plasma	100	ETL/ *n*-*i*-*p*	FTO/SnO_2_/100 °C/C_60_-SAM/FA_0.30_MA_0.70_PbI_3_/spiro-OMeTAD/Au	22.6	1.13	80.0	20.4	Toledo, 2017 [25]
SnO_2_	TDMASn + O_2_ Plasma	100	ETL/ *n*-*i*-*p*	PET/ITO/SnO_2_/C_60_-SAM/FA_0.30_MA_0.70_PbI_3_/spiro-OMeTAD/Au	22.1	1.10	75.4	18.4	Toledo, 2017 [33]
SnO_2_	TDMASn + O_2_ Plasma	100	ETL/ *n*-*i*-*p*	FTO/SnO_2_/C_60_-SAM/FA_0.30_MA_0.70_PbI_3_/spiro-OMeTAD/Au	21.6	1.13	78.1	19.0	Toledo, 2016 [34]
TiO_2_	TiCl_4_ + H_2_O	150	ETL/ *n*-*i*-*p*	FTO/TiO_2_ (17 nm)/*mp*-TiO_2_/MAPbI_3_/Graphene Oxide/spiro-OMeTAD/Au	20.2	1.04	73.0	15.1	Tsinghua, 2014 [35]
TiO_2_	TDMAT + H_2_O	120	ETL/ *n*-*i*-*p*	FTO/TiO_2_ (4 nm)/*mp*-TiO_2_/MAPbI_3_/spiro-OMeTAD/Au	23.1	1.08	73.4	18.3	Nanjing, 2018 [26]
TiO_2_	TDMAT + H_2_O	150	ETL/ *n*-*i*-*p*	ITO/TiO_2_ (10 nm)/*np*-SnO_2_/PC_61_BM/FA_0.30_MA_0.70_Pb(I_1-*x*_Cl*_x_*)_3_/spiro-OMeTAD/Au	23.0	1.08	78.2	19.5	Xidian, 2019 [36]
TiO_2_	TDMAT + H_2_O	225	ETL/ *n*-*i*-*p*	FTO/TiO_2_ (11 nm)/*mp*-TiO_2_/MAPbI_3_	22.3	1.11	74.0	18.4	Tokyo, 2019 [37]
TiO_2_	TDMAT + H_2_O		ETL/ *n*-*i*-*p*	FTO/*mp*-Sb:SnO_2_/TiO_2_ (10 nm)/MAPbI_3_/PTAA/Au	23.8	1.10	77.0	20.1	Soochow, 2018 [28]
TiO_2_	TDMAT + H_2_O	120	ETL, Passivation/ *n*-*i*-*p*	FTO/*np*-TiO_2_/TiO_2_ (2 nm)/MAPbI_3_/spiro-OMeTAD/Au	17.6	0.97	67.0	11.5	EPFL, 2014 [38]
TiO_2_	TTIP + O_2_ Plasma	130	ETL/ *n*-*i*-*p*	ITO/CF_4_ plasma TiO_2_ (20 nm)/MAPbI_3_/spiro-OMeTAD/Au	20.3	1.03	75.5	15.8	Eindhoven, 2018 [39]
TiO_2_	Ti(CpMe)(NMe_2_)_3_ + H_2_O	150	ETL Passivation/ *n*-*i*-*p*	ITO/ZnO (80 nm)/TiO_2_ (<3 nm)/Cs_0.15_FA_0.75_MA_0.10_PbI_2.9_Br_0.1_/spiro-OMeTAD/MoO_3_/Au	22.5	1.03	74.0	17.1	Soochow, 2018 [40]
TiO_2_	TTIP + H_2_O	250	Passivation/*n*-*i*-*p*	FTO/*c*-TiO_2_/*NR*-TiO_2_/TiO_2_ (4 nm)/MAPbI_3_/spiro-OMeTAD/Au	19.8	0.95	72.0	13.5	CNU, 2015 [41]
TiN	TiCl_4_ + NH_3_	350	ETL Passivation/ *n*-*i*-*p*	FTO/*c*-TiO_2_/*mp*-TiO_2_/TiN (<2 nm)/FA_0.83_MA_0.17_Pb(I_0.83_Br_0.17_)_3_/PTAA/Au	22.5	1.14	75.0	19.0	CNU, 2020 [42]
TiO_2_-IrO*_x_*	TDMAT + H_2_O (EtCp)Ir(CHD) + O_3_	175	HTL/ *p*-*i*-*n*	ITO/TiO_2_-IrO*_x_*(10 nm)/Cs_0.17_FA_0.83_Pb(I_0.83_Br_0.17_)_3_/C_60_/BCP/Ag	19.6	1.01	80.0	15.8	Stanford, 2018 [43]
GaN	TEG + Ar/N_2_/H_2_ plasma	280	ETL/ *n*-*i*-*p*	FTO/GaN (5 nm)/FA_0.85_MA_0.15_Pb(I_0.85_Br_0.15_)_3_/spiro-OMeTAD/Au	22.6	0.98	68.9	15.2	UST Beijing, 2019 [30]
HfO_2_	TEMAHf + H_2_O	90	Passivation/ *n*-*i*-*p*	PEN/ITO/HfO_2_ (<1 nm)/SnO_2_/Cs_0.05_(FA_0.83_MA_0.17_)_0.95_Pb(I_0.83_Br_0.17_)_3_ + RbI + KI/spiro-OMeTAD/Au	21.2	1.14	79.2	19.1	Xiamen, 2019 [44]
Nb_2_O_5_	(tert-butylimido)bis(diethylamino)niobium + O_3_	170	ETL/ *n*-*i*-*p*	FTO/Nb_2_O_5_ (15 nm)/FA_0.85_MA_0.15_Pb(I_0.85_Br_0.15_)_3_/spiro-OMeTAD/Au				Very low	EPFL, 2015 [31]
NiO	Ni(MeCp)_2_ + O_2_	350	HTL/ *p*-*i*-*n*	ITO/s-ALD NiO*_x_*/FA_0.2_MA_0.8_PbI_3_/PC_61_BM/Al	23.0	1.08	81.0	17.1	Cambridge, 2018 [45]
NiO	Ni(MeCp)_2_ + O_2_ plasma	150	HTL/ *p*-*i*-*n*	ITO/NiO (10 nm)/Cs_0.05_(FA_0.83_MA_0.17_)Pb(I_0.83_Br_0.17_)_3_/C_60_/BCP/Cu	21.8	1.07	73.4	17.1	Eindhoven, 2019 [46]
NiO, AZO, Al_2_O_3_	Ni(dmamb)_2_ + O_3_, TMA/DEZ + H_2_O	200, 100, 100	ETL/ *p*-*i*-*n*	FTO/NiO (6 nm)/Cs_0.05_MA_0.95_PbI_3_/PCBM/BCP/AZO (40 nm)/Ag/Al_2_O_3_ (50 nm)	22.5	1.03	80.8	18.8	SKKU, 2018 [47]
VO*_x_*	V(dma)_4_ + H_2_O	50	HTL/ *p*-*i*-*n*	ITO/VO*_x_* (1 nm)/MAPbI_3_/PC_61_BM/BCP/Ag	17.9	0.90	71.2	11.5	Peking, 2018 [48]
ZnO/Al_2_O_3_	DEZ + H_2_O	150	ETL/ *n*-*i*-*p*	FTO/ZnO (50 nm)/Al_2_O_3_ (<1 nm)/*mp*-TiO_2_/MAPbI_3_/spiro-OMeTAD/Au	18.9	1.01	62.0	15.6	UST Beijing, 2016 [49]
ZnS	DEZ + H_2_S	150	Passivation/ *n*-*i*-*p*	FTO/*c*-TiO_2_/*mp*-TiO_2_/ZnS (<2 nm)/FA_0.85_MA_0.15_Pb(I_0.85_Br_0.15_)_3_/PTAA/Au	22.5	1.13	75.0	18.8	CNU, 2020 [29]

**Table 2:** Summary of literature on ALD-grown layers above the absorber in perovskite solar cells.

Material	Precursors	Temp. (°C)	Application/Structure	Device Stack	*J_SC_* (mA/cm^2^)	*V_OC_* (V)	*FF* (%)	*η* (%)	Institute, Year [Ref]
Al_2_O_3_	TMA + H_2_O	100	Passivation/ *n*-*i*-*p*	ITO/*c*-TiO_2_/MAPb(I_1-*x*_Cl*_x_*)_3_/Al_2_O_3_ (1 nm)/spiro-OMeTAD/Au	21.7	1.07	77.0	18.0	Eindhoven, 2017 [62]
NiO, AZO, Al_2_O_3_	Ni(dmamb)_2_ + O_3_, TMA/DEZ + H_2_O	200, 100, 100	ETL/ *p*-*i*-*n*	FTO/NiO (6 nm)/Cs_0.05_MA_0.95_PbI_3_/PCBM/BCP/AZO (40 nm)/Ag/Al_2_O_3_ (50 nm)	22.5	1.03	80.8	18.8	SKKU, 2018 [47]
AZO	TMA/DEZ + H_2_O	85	Recombination/ *p*-*i*-*n*	ITO/PolyTPD/PFN/Cs_0.30_FA_0.60_MA_0.10_Pb(I_0.80_Br_0.20_)_3_/LiF/C_60_/PEIE/AZO (25 nm)/IZO/PEDOT:PSS/Cs_0.25_FA_0.75_Sn_0.5_Pb_0.5_I_3_/C_60_/BCP/Au	15.6	1.82	75.0	21.3	NREL, 2019 [63]
CuO*_x_*	Cu(dmamb)_2_ + H_2_O	100	Buffer/ *n*-*i*-*p* (ST)	FTO/*c*-TiO_2_/*mp*-TiO_2_/FA_0.95_MA_0.05_Pb(I_0.95_Br_0.05_)_3_/PTAA/pulsed-CVD CuO*_x_* (15 nm)/ITO	21.7	1.01	71.1	15.6	KRICT, 2020 [60]
CuO*_x_*	ATHFAACu + H_2_O	100	Buffer/ *n*-*i*-*p* (ST)	FTO/*c*-TiO_2_/*mp*-TiO_2_/Cs_0.05_(MA_0.17_FA_0.83_)_0.95_Pb(I_0.83_Br_0.17_)_3_/PTAA/AP-CVD CuO*_x_* (3 nm)/ITO/MgF_2_	20.6	1.10	73.7	16.7	Cambridge, 2020 [64]
Ga_2_O_3_	Ga_2_(NMe_2_)_6_ + H_2_O	120	Passivation/ *p*-*i*-*n*	FTO/Li:NiO/MAPbI_3_/IDIC/PCBM/BCP/Ga_2_O_3_ (<2 nm)/Ag	22.4	1.12	79.4	19.9	Wuhan, 2018 [65]
SnO_2_	TDMASn + H_2_O	100	Buffer/ *p*-*i*-*n* (2-T)	Si PV/ITO/PTAA/Cs_0.15_(FA_0.83_MA_0.17_)_0.85_Pb(I_0.7_Br_0.3_)_3_/ICBA/C_60_/SnO_2_/IZO/MgF_2_	17.8	1.80	79.4	25.4	UNC, 2019 [66]
SnO_2_	TDMASn + H_2_O	100	Buffer/ *p*-*i*-*n* (2-T)	Si PV/spiro-TTB/Cs*_x_*FA_1-*x*_Pb(I_1-*y*_Br*_y_*)_3_/LiF/C_60_/SnO_2_/IZO/MgF_2_	19.5	1.74	74.7	25.4	EPFL, 2019 [67]
SnO_2_	TDMASn + H_2_O		Buffer/ *p*-*i*-*n* (2-T)	Si PV/ITO/PTAA/Cs_0.05_(FA_0.83_MA_0.17_)_0.95_Pb(I_0.83_Br_0.17_)_3_/C_60_/SnO_2_ (20 nm)/IZO/AR foil	18.5	1.76	78.5	25.5	HZB, 2018 [68]
SnO*_x_*/Zn:SnO*_x_*	TDMASn/DEZ + H_2_O	85	Buffer/ *p*-*i*-*n* (ST)	ITO/PTAA/Cs_0.05_FA_0.80_MA_0.15_Pb (I_0.85_Br_0.15_)_3_/C_60_/BCP/SnO*_x_* (6 nm)/Zn:SnO*_x_* (2 nm)/IZO	20.8	1.12	79.3	18.5	NREL, 2019 [59]
SnO_2_/Zn:SnO*_x_*	TDMASn/DEZ + H_2_O	85	Buffer/ *p*-*i*-*n* (ST)	ITO/PTAA/Cs_0.15_FA_0.65_MA_0.20_Pb (I_0.80_Br_0.20_)_3_ + PEAI + Pb(SCN)_2_/C_60_/SnO*_x_* (6 nm)/Zn:SnO*_x_* (2 nm)/IZO	19.6	1.14	76.8	17.1	NREL, 2019 [69]
SnO_2_/Zn:SnO*_x_*	TDMASn/DEZ + H_2_O	90	ETL/ *p*-*i*-*n*	ITO/Poly-TPD/PFN/Cs_0.25_FA_0.75_Pb(I_0.80_Br_0.20_)_3_/LiF/C_60_/PEIE/SnO_2_/Zn:SnO*_x_*/Au	19.7	1.15	81.8	18.6	Stanford, 2019 [70]
TiO_2_	TDMAT + H_2_O	60	ETL/ *p*-*i*-*n*	ITO/NiO/MAPbI_3_/PC_61_BM (40 nm)/TiO_2_ (2 nm)/Ag	22.8	1.04	76.9	18.3	Nanjing, 2018 [71]
VO*_x_*	VTIP + H_2_O	80	Buffer/ *n*-*i*-*p* (ST)	ITO/*np*-SnO_2_/C_60_/FA_0.83_MA_0.17_Pb(I_0.83_Br_0.17_)_3_/spiro-TTB/VO*_x_* (9 nm)/ITO	18.9	1.07	71.0	14.2	Stanford, 2019 [72]
ZnO	DEZ + H_2_O	80	ETL/ *p*-*i*-*n*	ITO/PEDOT:PSS/MAPbI_3_/ZnO (40 nm)/Ag NWs/ALD Al_2_O_3_ (50 nm)-coated PET	20.7	1.02	76.4	16.2	Feng Chia, 2015 [73]
ZrO_2_	TDMAZr + O_3_	80	Passivation/ *p*-*i*-*n*	FTO/NiO*_x_*/*e*-MoO*_x_* (10 nm)/MAPbI_3_/ZrO_2_ (<2 nm)/PC_61_BM/Al	21.9	1.11	75.0	18.2	SCN, 2018 [74]

**Table 3:** Summary of advantages and disadvantages of conventional ALD and its variations.

Deposition Method	Advantages	Disadvantages
Conventional ALD	- Conformal pinhole-free uniform coating - Suitable for ultra-thin films	- Low growth rate - Low vacuum levels required
Pulsed-CVD	- Improved growth rates compared to conventional ALD	- Low vacuum levels required - Not suitable for ultra-thin films - Conformal coating not as good as conventional ALD
Spatial ALD	- Atmospheric pressure (no vacuum required) - Very fast growth rates.	- Sensitive to ambient oxygen/water when processed in air - Available precursors limited

## Data Availability

Data sharing not applicable.
